# Supplementary material for: The liver microenvironment orchestrates FGL1-mediated immune escape and progression of metastatic colorectal cancer
Source: Nat Commun. 2023 Oct 23;14:6690. doi: 10.1038/s41467-023-42332-0 (PMC10593839; doi:10.1038/s41467-023-42332-0)
Supplement: Supplementary file 1 — Supplementary Information [file 41467_2023_42332_MOESM1_ESM.pdf]

Supplementary information

The Liver Microenvironment Orchestrates FGL1-mediated Immune Escape and Progression of Metastatic Colorectal Cancer

Jia-Jun Li, Jin-Hong Wang, Tian Tian, Jia Liu, Yong-Qiang Zheng, Hai-Yu Mo, Hui Sheng, Yan-Xing Chen, Qi-Nian Wu, Yi Han, Kun Liao, Yi-Qian Pan, Zhao-Lei Zeng, Ze-Xian Liu, Wei Yang, Rui-Hua Xu, Huai-Qiang Ju

Supplementary information includes six Supplementary Figures.

Supplementary Figure 1. FGL1 facilitates the progression of colorectal cancer through immunosuppression in the liver microenvironment.

Supplementary Figure 2. High FGL1 levels predict poor outcomes and less benefit from PD-1/PD-L1 blockade therapy.

Supplementary Figure 3. TAMs promote the stabilization of FGL1 by activating NF-κB in the liver microenvironment.

Supplementary Figure 4. OTUD1 is transcriptionally upregulated by the TAM/NF-κB signaling pathway.

Supplementary Figure 5. The TAM/TNFα-OTUD1-FGL1 axis promotes immune escape and progression of CRLM.

Supplementary Figure 6. Benzethonium chloride inhibits tumor cell progression in the liver microenvironment by reducing the secretion of FGL1.

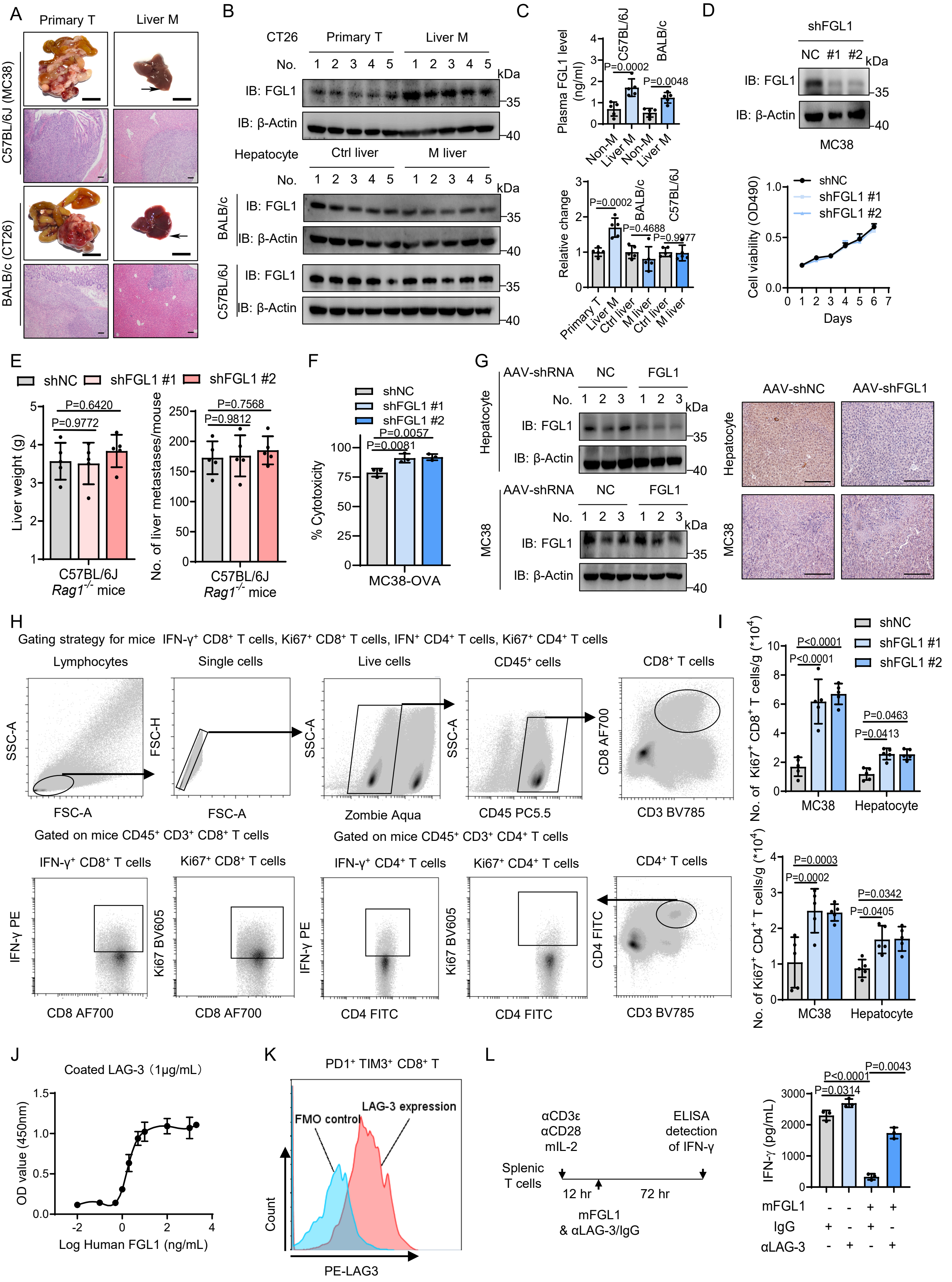
A
Primary T
Liver M
C57BL/6J (MC38)
BALB/c (CT26)
B
CT26
Primary T
Liver M
No. 1 2 3 4 5 1 2 3 4 5
kDa
IB: FGL1
35
IB: β-Actin
40
Hepatocyte
Ctrl liver
M liver
BALB/c
C57BL/6J
C
Plasma FGL1 level (ng/ml)
C57BL/6J
BALB/c
P=0.0002
P=0.0048
Non-M
Liver M
Relative change
P=0.0002
P=0.4688
P=0.9977
Primary T
Ctrl liver
M liver
D
shFGL1
NC #1 #2
IB: FGL1
IB: β-Actin
MC38
Cell viability (OD490)
shNC
shFGL1 #1
shFGL1 #2
Days
E
shNC shFGL1 #1 shFGL1 #2
Liver weight (g)
P=0.6420
P=0.9772
No. of liver metastases/mouse
P=0.7568
P=0.9812
C57BL/6J Rag1-/- mice
F
% Cytotoxicity
P=0.0057
P=0.0081
MC38-OVA
G
AAV-shRNA
NC
FGL1
Hepatocyte
MC38
AAV-shNC
AAV-shFGL1
H
Gating strategy for mice IFN-γ+ CD8+ T cells, Ki67+ CD8+ T cells, IFN+ CD4+ T cells, Ki67+ CD4+ T cells
Lymphocytes
Single cells
Live cells
CD45+ cells
CD8+ T cells
SSC-A
FSC-A
FSC-H
Zombie Aqua
CD45 PC5.5
CD8 AF700
CD3 BV785
Gated on mice CD45+ CD3+ CD8+ T cells
Gated on mice CD45+ CD3+ CD4+ T cells
IFN-γ+ CD8+ T cells
Ki67+ CD8+ T cells
IFN-γ+ CD4+ T cells
Ki67+ CD4+ T cells
CD4+ T cells
IFN-γ PE
Ki67 BV605
CD4 FITC
I
No. of Ki67+ CD8+ T cells/g (*10^4)
P<0.0001
P<0.0001
P=0.0463
P=0.0413
MC38
Hepatocyte
No. of Ki67+ CD4+ T cells/g (*10^4)
P=0.0003
P=0.0002
P=0.0342
P=0.0405
J
Coated LAG-3（1μg/mL）
OD value (450nm)
Log Human FGL1 (ng/mL)
K
PD1+ TIM3+ CD8+ T
FMO control
LAG-3 expression
Count
PE-LAG3
L
αCD3ε
αCD28
mIL-2
ELISA detection of IFN-γ
Splenic T cells
12 hr
72 hr
mFGL1 & αLAG-3/IgG
IFN-γ (pg/mL)
P<0.0001
P=0.0314
P=0.0043
mFGL1 - - + +
IgG + - + -
αLAG-3 - + - +

Supplementary Figure 1. FGL1 facilitates the progression of colorectal cancer through immunosuppression in the liver microenvironment. (A) Representative bright-field images and H&E staining of primary tumor and metastatic liver from C57BL/6J or BALB/c mice orthotopically implanted with MC38 or CT26 cells. Scale bar for bright-field images, 1 cm; scale bar for H&E images, 100 μm. (B) IB detection of FGL1 in CT26 cells from primary and liver metastatic tumor tissues, as well as in hepatocytes from control and metastatic livers. Right panel, quantitative estimates of FGL1 levels based on IB analyses. (C) Enzyme-linked immunosorbent assay (ELISA) detection of plasma FGL1 levels in mice grouped by the status of liver metastasis (n=5 mice per group). (D) Cell viability assay (MTS) analysis of the cell proliferation rates according to the optical density (OD490) of the indicated cells, n=3 biologically independent experiments. The knockdown of FGL1 expression was detected by IB. (E) Quantification of liver weight and the number of liver metastases in C57BL/6J *Rag1*$^{-/-}$ mice implanted intraportally with the control (shNC) and FGL1 knockdown MC38 cells (shFGL1 #1, shFGL1 #2) (n=5 mice per group). (F) Quantification of the indicated cells cocultured with activated OT-1 T cells for 8 hr and then subjected to the MTS assay, n=3 biologically independent experiments. The ratio of MC38-OVA cells to OT-1 T cells was 5:1. OVA, ovalbumin. (G) IB detection and IHC staining of FGL1 in hepatocytes or MC38 cells from mice treated with AAV-shNC or AAV-shFGL1. Scale bar, 100 μm. (H) Gating strategy for the flow cytometric analysis. (I) Flow cytometric analysis of the number of Ki67$^+$ CD8$^+$ or Ki67$^+$ CD4$^+$ T cells in liver metastases from mice with knockdown of FGL1 in MC38 cells or hepatocytes, respectively (n=5 mice per group). (J) Binding curve of recombinant hFGL1 on recombinant hLAG-3, n=3 biologically independent experiments. (K) Flow cytometric analysis of LAG-3 expression on PD1$^+$ TIM3$^+$ CD8$^+$ T cells from liver metastases. The Fluorescence Minus One (FMO) control was used to determine LAG-3 positivity. (L) ELISA detection of IFN-γ secreted from splenic T cells treated as indicated, n=3 biologically independent experiments. IB experiments in B, D, G were repeated three times, the data are representative of three biologically independent experiments. The data in B, C, D, E, F, I, J, L are presented as the mean $\pm$ SD. *P* values were determined by one-way ANOVA (B, C, E, F, I, L).

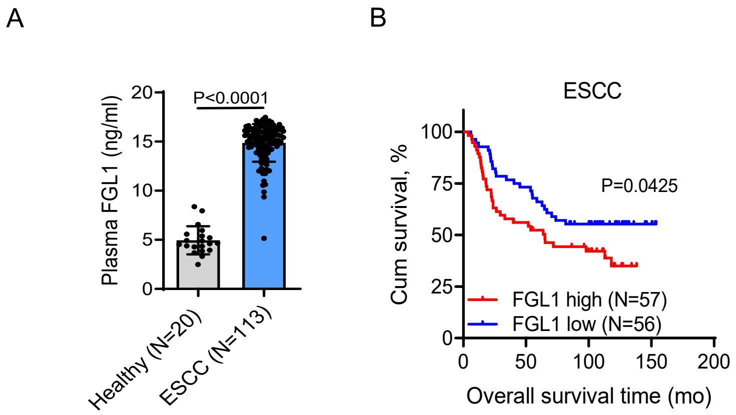

Supplementary Figure 2. High FGL1 levels predict poor outcomes and less benefit from PD-1/PD-L1 blockade therapy. (A) ELISA detection of plasma FGL1 levels in healthy donor (N=20) and ESCC (N=113) specimens (SYSUCC cohort). (B) Kaplan–Meier analysis of the overall survival of ESCC patients (N=113, SYSUCC cohort) with plasma FGL1 levels detected in (A). The data in A are presented as the mean ± SD. The data in B were determined by Kaplan–Meier analysis with the log-rank test. *P* values were determined by two-tailed unpaired Student's *t* test (A).

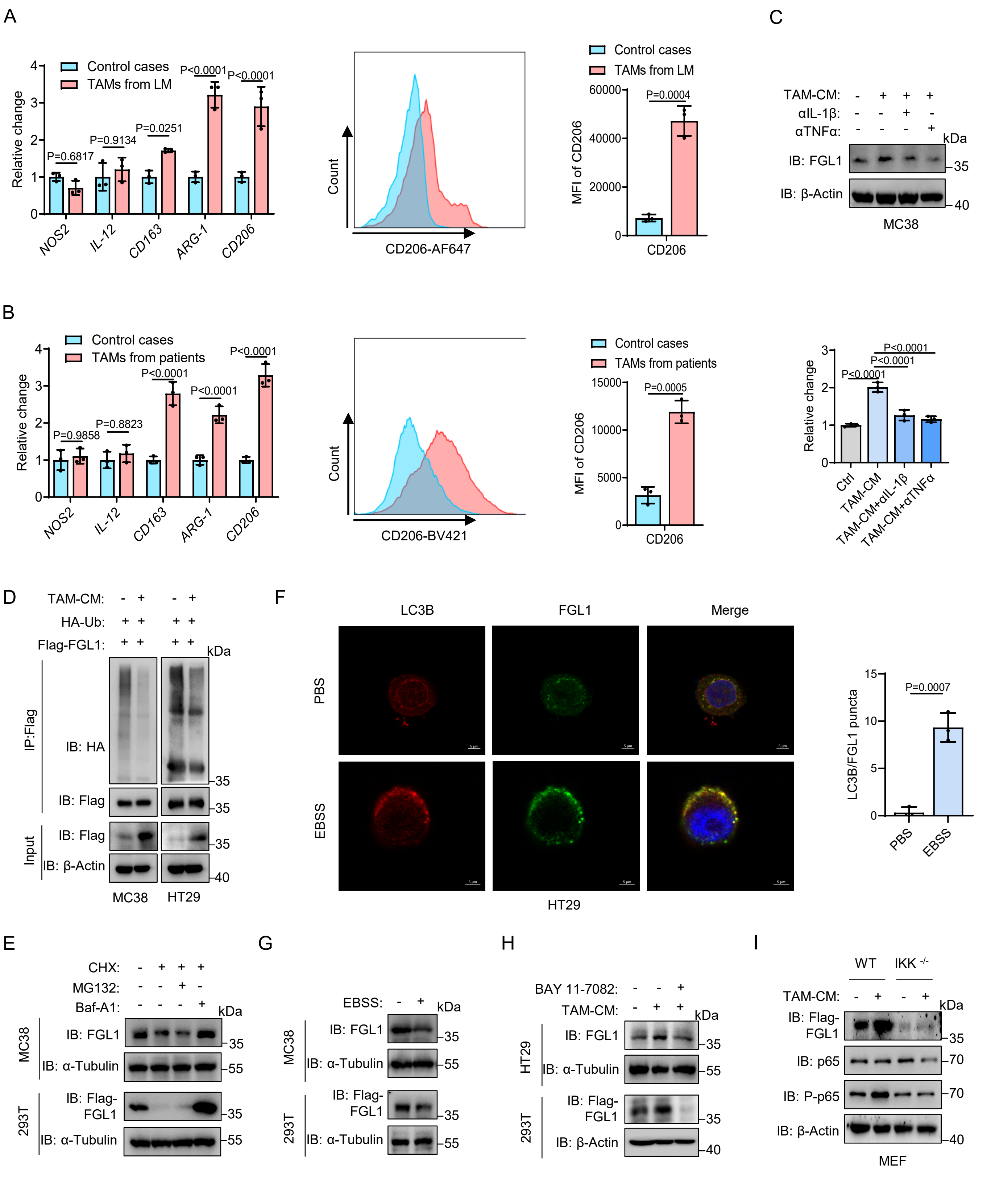

Supplementary Figure 3. TAMs promote the stabilization of FGL1 by activating NF-κB in the liver microenvironment. (A) qPCR and flow cytometric analysis of the markers of the TAMs from different mouse liver metastases and control cases, n=3 biologically independent experiments. (B) qPCR and flow cytometric analysis of the markers of the TAMs from different CRLM patients and control cases, n=3 biologically independent experiments. (C) IB detection and quantification of FGL1

expression in MC38 cells incubated with conditioned media produced by TAMs (TAM-CM) and treated with neutralization antibodies for IL-1β and TNFα, n=3 biologically independent experiments. (D) Co-IP analysis of the interaction between HA-Ub and Flag-FGL1 in MC38 and HT29 cells incubated with TAM-CM. (E) IB detection of FGL1 expression in MC38 cells and 293T cells treated with MG132 (10 μM, 12 hr) or bafilomycin A1 (100 nM, 24 hr) and subjected to cycloheximide (CHX, 40 μg/mL) exposure for 1 hr. (F) Confocal microscopic analysis and quantification of the colocalization of FGL1 and LC3B in HT29 cells treated with EBSS for 0.5 hr, n=3 biologically independent experiments. Scale bar, 5 μm. (G) IB detection of FGL1 expression in MC38 or 293T cells treated with Earle's balanced salt solution (EBSS) for 0.5 hr. (H) IB detection of FGL1 expression in HT29 and 293T cells pretreated with BAY 11-7082 (10 μM) for 1 hr followed by incubation with TAM-CM for 16 hr. (I) IB detection of Flag-FGL1, p65 and P-p65 expression in WT or $IKK^{-/-}$ MEFs transiently overexpressing Flag-FGL1 following incubation with TAM-CM for 16 hr. IB experiments in C-E, G-I were repeated three times, the data are representative of three biologically independent experiments. The data in A, B, C, F are presented as the mean ± SD. The *P* value was determined by two-tailed unpaired Student's *t* test (A, B, F) and one-way ANOVA (A, B, C).

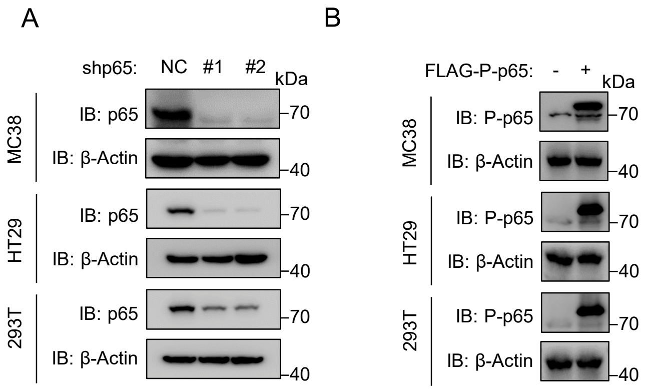

Supplementary Figure 4. OTUD1 is transcriptionally upregulated by the TAM/NF-κB signaling pathway. (A) IB detection of p65 expression in the indicated cells. (B) IB detection of P-p65 expression in the indicated cells. IB experiments in A-B were repeated three times, the data are representative of three biologically independent experiments.

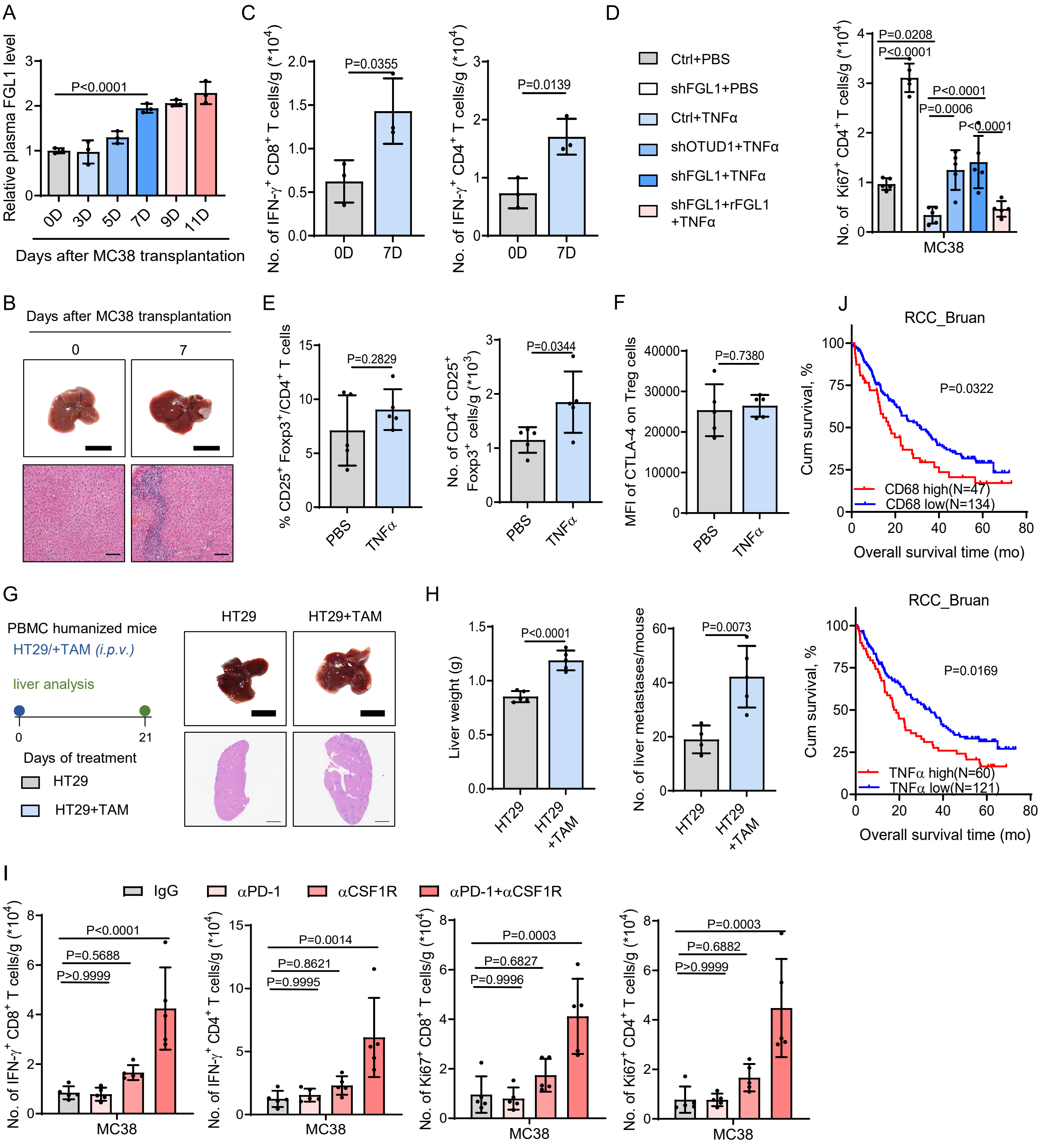

Supplementary Figure 5. The TAM/TNFα-OTUD1-FGL1 axis promotes immune escape and progression of CRLM. (A) ELISA detection of plasma FGL1 levels in mice treated as indicated (n=3 mice per group). (B) Representative H&E staining of liver tissues from mice treated as indicated. Scale bar for bright-field images, 1 cm; scale bar for H&E images, 100 μm. (C) Flow cytometric analysis of the number of IFN-γ⁺ CD8⁺, IFN-γ⁺ CD4⁺ T cells in liver metastases or normal liver from mice intraportally transplanted with MC38 cells for indicated times (n=3 mice per group). (D) Flow cytometric analysis of the number of Ki67⁺ CD4⁺ T cells in liver metastases from mice of indicated

groups (n=5 mice per group). (E) Flow cytometric analysis of the frequency and the number of $CD4^+$ $CD25^+$ $Foxp3^+$ cells within $CD4^+$ T cells in liver metastases from mice treated as indicated (n=5 mice per group). (F) Flow cytometric analysis of CTLA-4 expression on Treg cells within liver metastases from mice treated as indicated (n=5 mice per group). (G) Representative H&E staining of liver metastases from PBMC-humanized mice implanted with HT29/+TAM cells via intraportal vein injection. Scale bar for bright-field images, 1 cm; scale bar for H&E images, 1.25 mm. (H) Quantification of liver weight and the number of liver metastases of indicated groups of mice (n=5 mice per group). (I) Flow cytometric analysis of the number of IFN-γ^+ $CD8^+$, IFN-γ^+ $CD4^+$, $Ki67^+$ $CD8^+$ and $Ki67^+$ $CD4^+$ T cells in liver metastases from mice treated with the control (IgG), anti-CSF1R and/or anti-PD-1 monoclonal antibodies (n=5 mice per group). (J) CD68 (N=181) or TNFα (N=181) expression is inversely correlated with overall survival in patients treated with anti-PD-1 in the study of Braun and colleagues on renal cell carcinoma. The data in A, C, D-F, H-I are presented as the mean $\pm$ SD. The data in J were determined by Kaplan–Meier analysis with the log-rank test. *P* values were determined by one-way ANOVA (A, D, I) and two-tailed unpaired Student's *t* test (C, E, F, H).

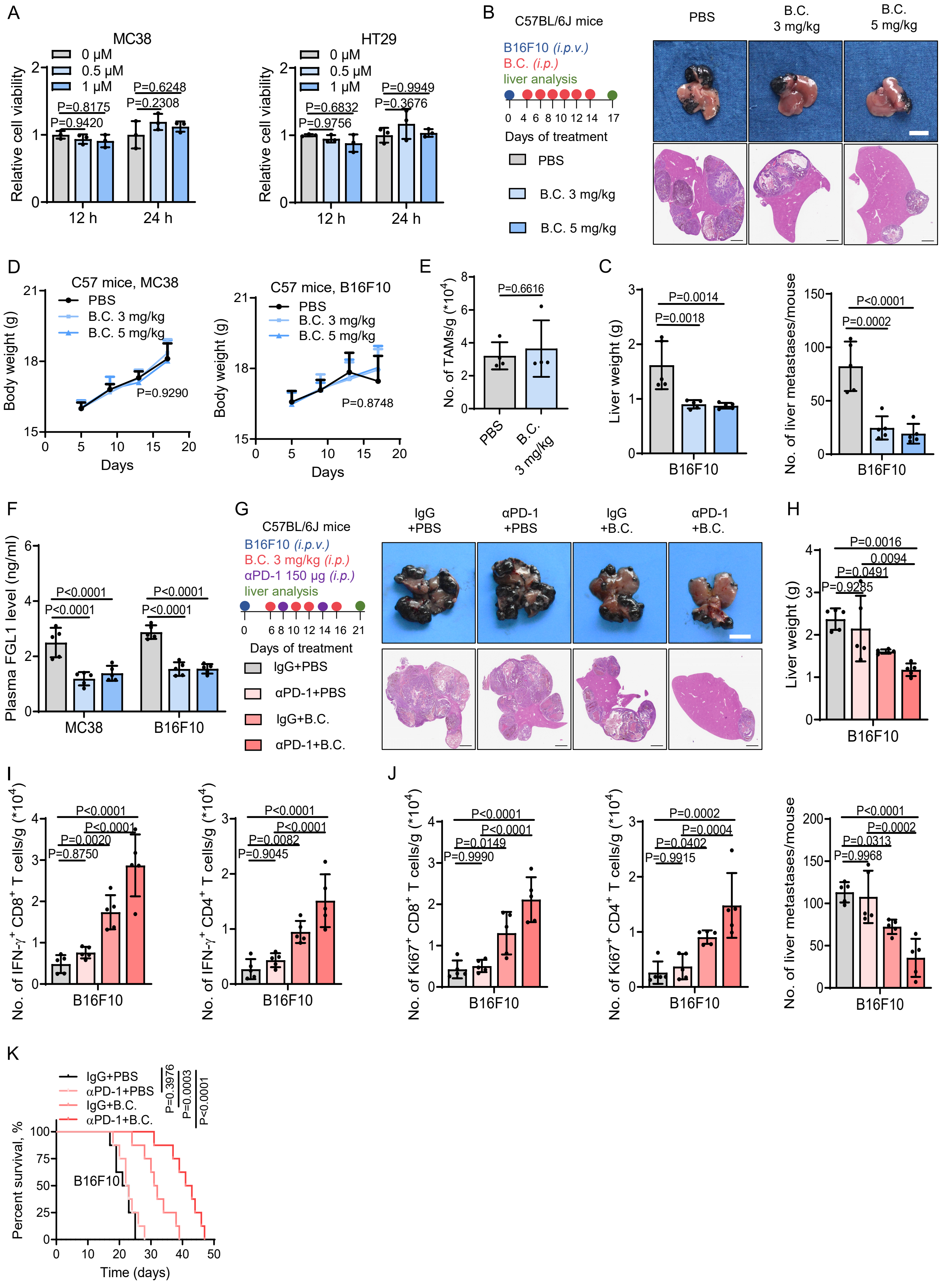

A
MC38
0 μM
0.5 μM
1 μM
Relative cell viability
P=0.8175
P=0.9420
P=0.6248
P=0.2308
12 h
24 h
HT29
P=0.6832
P=0.9756
P=0.9949
P=0.3676
B
C57BL/6J mice
B16F10 (i.p.v.)
B.C. (i.p.)
liver analysis
0 4 6 8 10 12 14 17
Days of treatment
PBS
B.C. 3 mg/kg
B.C. 5 mg/kg
D
C57 mice, MC38
Body weight (g)
P=0.9290
Days
C57 mice, B16F10
P=0.8748
E
No. of TAMs/g (*10^4)
P=0.6616
PBS
B.C. 3 mg/kg
C
Liver weight (g)
P=0.0014
P=0.0018
B16F10
No. of liver metastases/mouse
P<0.0001
P=0.0002
F
Plasma FGL1 level (ng/ml)
P<0.0001
MC38
B16F10
G
C57BL/6J mice
B16F10 (i.p.v.)
B.C. 3 mg/kg (i.p.)
αPD-1 150 μg (i.p.)
liver analysis
0 6 8 10 12 14 16 21
Days of treatment
IgG+PBS
αPD-1+PBS
IgG+B.C.
αPD-1+B.C.
H
Liver weight (g)
P=0.0016
0.0094
P=0.0491
P=0.9285
I
No. of IFN-γ+ CD8+ T cells/g (*10^4)
P<0.0001
P=0.0020
P=0.8750
No. of IFN-γ+ CD4+ T cells/g (*10^4)
P=0.0082
P=0.9045
J
No. of Ki67+ CD8+ T cells/g (*10^4)
P=0.0149
P=0.9990
No. of Ki67+ CD4+ T cells/g (*10^4)
P=0.0002
P=0.0004
P=0.0402
P=0.9915
No. of liver metastases/mouse
P=0.0313
P=0.9968
K
P=0.3976
P=0.0003
P<0.0001
Percent survival, %
Time (days)

Supplementary Figure 6. Benzethonium chloride inhibits tumor cell progression in the liver microenvironment by reducing the secretion of FGL1. (A) Cell viability assay (MTS) analysis of MC38 cells and HT29 cells treated with indicated doses of benzethonium chloride for 12 hr or 24 hr, n=3 biologically independent experiments. (B) Representative H&E staining of metastatic livers from C57BL/6J mice implanted with B16F10 cells via portal vein injection followed by treatment with PBS or benzethonium chloride as indicated. Scale bar for bright-field images, 1 cm; scale bar for H&E images, 2.5 mm. B.C., benzethonium chloride. (C) Quantification of liver weight and the number of liver metastases of mice treated with PBS, 3 mg/kg or 5 mg/kg benzethonium chloride (n=5 mice per group). (D) Body weight of mice treated with PBS, 3 mg/kg or 5 mg/kg benzethonium chloride (n=5 mice per group). (E) Flow cytometric analysis of the number of TAMs in liver metastases from mice treated with PBS or benzethonium chloride (3mg/kg) (n=4 mice per group). (F) ELISA detection of plasma FGL1 levels in mice implanted with MC38 cells or B16F10 cells via portal vein injection followed by treatment with PBS or benzethonium chloride (3 mg/kg, 5 mg/kg) (n=5 mice per group). (G) Representative H&E staining of metastatic livers from C57BL/6J mice implanted with B16F10 cells via portal vein injection followed by treatment with the control (IgG), benzethonium chloride and/or anti-PD-1 monoclonal antibody as indicated. Scale bar for bright-field images, 1 cm; scale bar for H&E images, 2.5 mm. (H) Quantification of liver weight and the number of liver metastases of the indicated groups of mice (n=5 mice per group). (I-J) Flow cytometric analysis of the number of IFN-γ^+ $CD8^+$, IFN-γ^+ $CD4^+$ (I), Ki67$^+$ $CD8^+$ and Ki67$^+$ $CD4^+$ T cells (J) in liver metastases in the indicated groups of mice (n=5 mice per group). (K) Survival curve analysis of mice implanted with B16F10 cells via portal vein injection followed by treatment as indicated (Mean survival times of IgG+PBS, αPD-1+PBS, IgG+B.C. and αPD-1+B.C. mice were 21.5, 22.9, 32 and 41 days, respectively) (n=8 mice per group). The data in A, C, E, F, H-J are presented as the mean ± SD. *P* values were determined by two-way ANOVA (D), one-way ANOVA (A, C, F, H-J) and two-tailed unpaired Student's *t* test (E).
