# Supplementary material for: The liver microenvironment orchestrates FGL1-mediated immune escape and progression of metastatic colorectal cancer
Source: Nat Commun. 2023 Oct 23;14:6690. doi: 10.1038/s41467-023-42332-0 (PMC10593839; doi:10.1038/s41467-023-42332-0)
Supplement: Supplementary file 2 — Description of Additional Supplementary Files [file 41467_2023_42332_MOESM2_ESM.pdf]

Description of Additional Supplementary Files

File Name: **Supplementary Data 1**

Description: Details of FGL1 immunoprecipitation mass spectrum analysis.

File Name: **Supplementary Data 2**

Description: Details of oligonucleotides and primers used in this study.

File Name: **Supplementary Data 3**

Description: Details of all the antibodies, chemicals, peptides, recombinant proteins, critical commercial assays and software used in this study.
