# Supplementary material for: The liver microenvironment orchestrates FGL1-mediated immune escape and progression of metastatic colorectal cancer
Source: Nat Commun. 2023 Oct 23;14:6690. doi: 10.1038/s41467-023-42332-0 (PMC10593839; doi:10.1038/s41467-023-42332-0)
Supplement: Supplementary file 6 — Reporting Summary [file 41467_2023_42332_MOESM6_ESM.pdf]

nature portfolio

Corresponding author(s): Huai-Qiang Ju

Last updated by author(s): Sep 27, 2023

Reporting Summary

Nature Portfolio wishes to improve the reproducibility of the work that we publish. This form provides structure for consistency and transparency in reporting. For further information on Nature Portfolio policies, see our Editorial Policies and the Editorial Policy Checklist.

Statistics

For all statistical analyses, confirm that the following items are present in the figure legend, table legend, main text, or Methods section.

n/a	Confirmed	
☐	☒	The exact sample size (n) for each experimental group/condition, given as a discrete number and unit of measurement
☐	☒	A statement on whether measurements were taken from distinct samples or whether the same sample was measured repeatedly
☐	☒	The statistical test(s) used AND whether they are one- or two-sided<br>*Only common tests should be described solely by name; describe more complex techniques in the Methods section.*
☒	☐	A description of all covariates tested
☐	☒	A description of any assumptions or corrections, such as tests of normality and adjustment for multiple comparisons
☐	☒	A full description of the statistical parameters including central tendency (e.g. means) or other basic estimates (e.g. regression coefficient) AND variation (e.g. standard deviation) or associated estimates of uncertainty (e.g. confidence intervals)
☐	☒	For null hypothesis testing, the test statistic (e.g. F, t, r) with confidence intervals, effect sizes, degrees of freedom and P value noted<br>*Give P values as exact values whenever suitable.*
☒	☐	For Bayesian analysis, information on the choice of priors and Markov chain Monte Carlo settings
☒	☐	For hierarchical and complex designs, identification of the appropriate level for tests and full reporting of outcomes
☐	☒	Estimates of effect sizes (e.g. Cohen's d, Pearson's r), indicating how they were calculated

Our web collection on statistics for biologists contains articles on many of the points above.

Software and code

Policy information about availability of computer code

Data collection	Roche LightCycler 480 System (Roche), CytoFLEX LX (Beckman Coulter), ZEISS LSM 880 LSM880 confocal microscope equipped with fast airyscan (Carl Zeiss AG), ChemiDoc Touch Imaging System (Bio-Rad), IVIS Spectrum In Vivo Imaging System (PerkinElmer), Spark 10M Multimode Microplate Reader (TECAN), and Q Exactive HF-X mass spectrometer coupled to Easy nLC1200 (Thermo Scientific).
Data analysis	Image Lab version 4.1 (Bio-Rad) was used to acquire Immunoblots and protein Coomassie staining gels. Acquired raw images were analyzed using Image J 1.8.0 (https://imagej.nih.gov/ij), ZEISS ZEN 2.6 blue edition software (Carl Zeiss AG) and Living image software (PerkinElmer). Flow Cytometry data was analyzed using CytExpert software (Beckman Coulter) and FlowJo version 10; JASPAR software was used to predict the p53 binding sequence. The Single-cell transcriptome analyses were performed using Cellranger (4.0.0), Seurat (4.3.0), harmony (0.1.0), ggplot2 (3.3.5), dplyr (1.0.5). Statistical analysis was performed with R program version 4.0.3 (R Foundation for Statistical Computing). Statistical analysis was performed with GraphPad Prism version 8.0.2 (GraphPad Software, Inc.). The MS data were analyzed using MaxQuant software version 1.6.1.0.

For manuscripts utilizing custom algorithms or software that are central to the research but not yet described in published literature, software must be made available to editors and reviewers. We strongly encourage code deposition in a community repository (e.g. GitHub). See the Nature Portfolio guidelines for submitting code & software for further information.

Data

Policy information about availability of data

All manuscripts must include a data availability statement. This statement should provide the following information, where applicable:

- Accession codes, unique identifiers, or web links for publicly available datasets
- A description of any restrictions on data availability
- For clinical datasets or third party data, please ensure that the statement adheres to our policy

This study analyzes existing, publicly available data. These accession numbers are available from GEO under following accession codes: GSE164522 [https://www.ncbi.nlm.nih.gov/geo/query/acc.cgi?acc=GSE164522] and GSE31477 [https://www.ncbi.nlm.nih.gov/geo/query/acc.cgi?acc=GSE31477]. And cohorts for estimating the association between gene expression and prognosis after immunotherapy are sourced from Braun et al., 2020; Mariathasan et al., 2018; Riaz et al., 2017 and Snyder et al., 2017. Correlation between p65 and OTUD1 expression in CRC and GC were performed using data collected from TCGA (https://www.cancer.gov/about-nci/organization/ccg/research/structural-genomics/tcga) and CCLE (https://depmap.org/portal/download/all/). All mass spectrometry data are deposited in https://doi.org/10.6084/m9.figshare.24104007. The remaining data are available within the article, Supplementary information and Source data file. Source data are provided with this paper.

Research involving human participants, their data, or biological material

Policy information about studies with human participants or human data. See also policy information about sex, gender (identity/presentation), and sexual orientation and race, ethnicity and racism.

Reporting on sex and gender	The patients included males and females, and the information on sex and gender was not relevant in our study.
Reporting on race, ethnicity, or other socially relevant groupings	N/A
Population characteristics	A total of 296 CRC (110 female, 186 male, average age: 55 ± 11 years), 411 GC (130 female, 281 male, average age: 59 ± 13 years), 144 ESCC (39 female, 105 male, average age: 57 ± 8 years) and 52 NPC (6 female, 46 male, average age: 46 ± 11 years) patient samples were obtained from Sun Yat-sen University Cancer Center (SYSUCC, Guangzhou, China) between 2003 and 2022.
Recruitment	All CRC, GC, ESCC, NPC patient samples were obtained from Sun Yat-sen University Cancer Center (SYSUCC, Guangzhou, China) after appropriate review of our research project to ensure that it was covered under their ethics approval (2022112917400003OO289). All patient samples were collected by SUSYCC based on availability and informed consent provided by the individuals. Baseline characteristic of samples were balanced among groups without bias.
Ethics oversight	The study protocol was approved by the Institutional Review Board of Sun Yat-sen University Cancer Center (2022112917400003OO289). Written informed consent was obtained from the patients who provided samples.

Note that full information on the approval of the study protocol must also be provided in the manuscript.

Field-specific reporting

Please select the one below that is the best fit for your research. If you are not sure, read the appropriate sections before making your selection.

☒ Life sciences ☐ Behavioural & social sciences ☐ Ecological, evolutionary & environmental sciences

For a reference copy of the document with all sections, see nature.com/documents/nr-reporting-summary-flat.pdf

Life sciences study design

All studies must disclose on these points even when the disclosure is negative.

Sample size	No statistical method was used to predetermine sample size. Sample size was chosen based on previous experience and standards in the field (KLi, N., Quan, A., Li, D. et al. Nat Commun 14, 1986 (2023); or LWang, Y., Zhou, SK., Wang, Y. et al. Nat Commun 14, 1993 (2023).). For example, all of the experiments were repeated at least 3 times, and the sample sizes for in vitro (n >= 3) and in vivo (n >= 5) .
Data exclusions	No data were excluded for analysis.
Replication	For each representative image/data, experiments were performed at least three times with similar results unless otherwise noted in the manuscript.
Randomization	All the samples, organisms and participants were randomly allocated into different experimental groups.
Blinding	Investigators were not blinded to treatments due to the unbiased nature of molecular biological assays used, but no subjective assessments were made.

Reporting for specific materials, systems and methods

We require information from authors about some types of materials, experimental systems and methods used in many studies. Here, indicate whether each material, system or method listed is relevant to your study. If you are not sure if a list item applies to your research, read the appropriate section before selecting a response.

Materials & experimental systems

n/a	Involved in the study
☐	☒ Antibodies
☐	☒ Eukaryotic cell lines
☒	☐ Palaeontology and archaeology
☐	☒ Animals and other organisms
☒	☐ Clinical data
☒	☐ Dual use research of concern
☒	☐ Plants

Methods

n/a	Involved in the study
☒	☐ ChIP-seq
☐	☒ Flow cytometry
☒	☐ MRI-based neuroimaging

Antibodies

Antibodies used

Immunoblotting (dilution 1:1000 for all primary antibodies and 1:5000 for secondary antibodies)
Anti-human FGL1 antibody Abcam, Cat# ab170922.
Anti-mouse FGL1 antibody Novus, Cat# NBP2-16486
Anti-mouse Nectin 2 antibody Abcam, Cat# ab135246
Anti-mouse HMGB1 antibody Abcam, Cat# ab79823
Anti-mouse Gal-9 antibody Abcam, Cat# ab275877
Anti-mouse PD-L1 antibody Abcam, Cat# ab213480
Anti-mouse PD-L2 antibody Abcam, Cat# ab21107
Anti-mouse VISTA antibody CST, Cat# 54979
Anti-NF-κB p65 antibody CST, Cat# 8242S
Anti-Phospho-NF-κB p65 (Ser536) CST, Cat# 3033S
Anti-HA-Tag antibody DIA-AN, Cat# 2063
Anti-Flag-Tag antibody DIA-AN, Cat# 2064
Anti-Myc-Tag antibody DIA-AN, Cat# 2097
Anti-GST-Tag antibody CST, Cat# 2624S
Anti-human OTUD1 antibody Abcam, Cat# ab122481
Anti-mouse OTUD1 antibody MyBiosource, Cat# MBS3218844
Anti-Ubiquitin antibody CST, Cat# 3936S
Anti-β-Actin antibody CST, Cat# 4970S
Anti-α-Tubulin antibody CST, Cat# 2125S
Anti-mouse IgG antibody CST, Cat# 7076S
Anti-rabbit IgG antibody CST, Cat# 7074S

Immunohistochemistry
Anti-human FGL1 antibody Abcam, Cat# ab170922, 1:200
Anti-mouse FGL1 antibody Proteintech, Cat# 16000-1-AP, 1:200
Anti-human OTUD1 antibody Abcam, Cat# ab122481
Anti-human CD68 antibody ZSGB-Bio, Cat# ZM-0060, working solution
Anti-human CD4 antibody ZSGB-Bio, Cat# ZA-0519, working solution
Anti-human CD8 antibody ZSGB-Bio, Cat# ZA-0508, working solution

Immunofluorescence (dilution 1:100 for all antibodies)
Anti-human LC3B antibody Novus, Cat# NB100-2220
Anti-Flag-Tag antibody DIA-AN, Cat# 2064

Immunoprecipitation, 5 μg antibody/sample
Anti-Flag-Tag antibody DIA-AN, Cat# 2064
Anti-Myc-Tag antibody DIA-AN, Cat# 2097
Normal mouse IgG Santa Cruz, Cat# sc-2025

Chromatin Immunoprecipitation, 5 μg antibody/sample
Anti-NF-κB p65 antibody CST, Cat# 8242S
Normal rabbit IgG CST, Cat# 2729S

Flow cytometry (dilution 1:100 for all antibodies)
Brilliant Violet 421 anti-human CD206 antibody Biolegend, Clone 15-2, Cat# 321125

PerCP/Cyanine5.5 anti-mouse CD45 antibody Biolegend, Clone 30-F11, Cat# 103132
Brilliant Violet 785 anti-mouse CD3 antibody Biolegend, Clone 17A2, Cat# 100232
FITC anti-mouse CD4 antibody Biolegend, Clone RM4-5, Cat# 100509
Alexa Fluor® 700 anti-mouse CD8 antibody Biolegend, Clone 53-6.7, Cat# 100730
PE anti-mouse IFN-γ antibody Biolegend, Clone XMG1.2, Cat# 505808
Brilliant Violet 605™ anti-mouse Ki67 antibody Biolegend, Clone 16A8, Cat# 652413
PE anti-mouse F4/80 antibody Biolegend, Clone BM8, Cat# 123110
PE/Cyanine7 anti-mouse CD11b antibody Biolegend, Clone M1/70, Cat# 101216
Alexa Fluor® 647 anti-mouse CD206 antibody Biolegend, Clone C068C2, Cat# 141712
Brilliant Violet 605™ anti-mouse PD-1 antibody Biolegend, Clone 29F.1A12, Cat# 135220
PE/Cyanine7 anti-mouse TIM3 antibody Biolegend, Clone RMT3-23, Cat# 119716
PE anti-mouse LAG-3 antibody Biolegend, Clone C9B7W, Cat# 125208
Brilliant Violet 421™ anti-mouse CD25 antibody Biolegend, Clone PC61, Cat# 102043
Alexa Fluor® 647 anti-mouse Foxp3 antibody Biolegend, Clone 150D, Cat# 320014
PE anti-mouse CD152 antibody Biolegend, Clone UC10-4B9, Cat# 106306
Zombie Aqua Biolegend, Cat# 423102

Activation
Anti-mouse CD3ε antibody, functional grade Biolegend, Clone 145-2C11, Cat# 100340, 2.5 μg/mL
Anti-mouse CD28 antibody, functional grade Biolegend, Clone 37.51, Cat# 102116, 1.25 μg/mL

Neutralization
Anti-mouse IL-1 beta antibody Thermo Scientific, Clone B122, Cat# 14-7012-81, 1:200
Anti-mouse LAG-3 antibody BioXcell, Clone C9B7W, Cat# BP0174, 1:200
Anti-mouse TNFα antibody Biolegend, Clone TN3-19.12, Cat# 506111, 1:200
Rat IgG2a isotype control Bioxcell, Cat# BE0089, 1:200 or 400 μg
Anti-mouse CSF1R antibody Bioxcell, Clone AFS98, Cat# BE0213, 400 μg
Anti-mouse PD-1 antibody Junshi Biosciences, N/A, 150 μg
Mouse IgG1 isotype control Junshi Biosciences, N/A, 150 μg

Validation

Anti-human FGL1 antibody Abcam, Cat# ab170922. Supplier validation: "Species reactivity, Reacts with: Human, applications Suitable for: WB, IP".
Anti-mouse FGL1 antibody Novus, Cat# NBP2-16486. Supplier validation: "Applications: Immunohistochemistry, Western Blot; Reactivity Notes: Immunogen displays the following percentage of sequence identity for non-tested species: Rat (86%), Mouse (86%)".
Anti-mouse Nectin 2 antibody Abcam, Cat# ab135246. Supplier validation: "Reacts with: Mouse, Rat, Human, Suitable for: Flow Cyt (Intra), WB, ICC/IF, IP".
Anti-mouse HMGB1 antibody Abcam, Cat# ab79823. Supplier validation: "Reacts with: Mouse, Rat, Human, Suitable for: Flow Cyt (Intra), WB, ICC/IF, IP".
Anti-mouse Gal-9 antibody Abcam, Cat# ab275877. Supplier validation: "Reacts with: Mouse, Suitable for: ICC/IF, WB, Flow Cyt (Intra)".
Anti-mouse PD-L1 antibody Abcam, Cat# ab213480. Supplier validation: "Reacts with: Mouse, Suitable for: ICC/IF, IP, WB".
Anti-mouse PD-L2 antibody Abcam, Cat# ab21107. Supplier validation: "Reacts with: Mouse, Suitable for: IHC-P, Flow Cyt, WB, IP, Blocking, IHC-Fr".
Anti-mouse VISTA antibody CST, Cat# 54979. Supplier validation: " Reactivity, Human, Mouse, Rat, Suitable for: WB, IP, IHC, IF".
Anti-NF-κB p65 antibody CST, Cat# 8242S. Supplier validation: "Reactivity, Human, Mouse, Rat, Hm, Mk, Dg, Suitable for: WB, IP, IHC, IF, F, ChIP, C&R".
Anti-Phospho-NF-κB p65 (Ser536) CST, Cat# 3033S. Supplier validation: "Reactivity, Human, Mouse, Rat, Hm, Mk, Dg, Suitable for: WB, IP, IF, F".
Anti-HA-Tag antibody DIA-AN, Cat# 2063. Supplier validation: "Reactivity: Protein fused with HA tag, Application: ELISA, WB, IP, IF".
Anti-Flag-Tag antibody DIA-AN, Cat# 2064. Supplier validation: "Reactivity: Human,Mouse,Rat,Zebra fish, Application: ELISA, WB, IP, IF,ChIP".
Anti-Myc-Tag antibody DIA-AN, Cat# 2097. Supplier validation: " Reactivity: Protein fused with MYC tag, Application: ELISA, WB, IP, IF".
Anti-GST-Tag antibody CST, Cat# 2624S. Supplier validation: " Reactivity: All, Suitable for: WB, IP, IF".
Anti-human OTUD1 antibody Abcam, Cat# ab122481. Supplier validation: "Reacts with: Human, Suitable for: IHC-P".
Anti-mouse OTUD1 antibody MyBiosource, Cat# MBS3218844. Supplier validation: "Reactivity, Cow, Dog, Horse, Human, Mouse, Rat, Applications, WB".
Anti-Ubiquitin antibody CST, Cat# 3936S. Supplier validation: " Reactivity: All, Suitable for: WB, IHC".
Anti-β-Actin antibody CST, Cat# 4970S. Supplier validation: "Reactivity, Human, Mouse, Rat, Hm, Mk, Dg, Suitable for: WB, IHC, IF, F".
Anti-α-Tubulin antibody CST, Cat# 2125S. Supplier validation: "Reactivity, Human, Mouse, Rat, Dm, Mk, Pg, Suitable for: WB, IHC, IF, F".
Anti-mouse IgG antibody CST, Cat# 7076S. Supplier validation: "Reactivity, Mouse, Suitable for: WB".
Anti-rabbit IgG antibody CST, Cat# 7074S. Supplier validation: "Reactivity, Rabbit, Suitable for: WB".
Anti-mouse FGL1 antibody Proteintech, Cat# 16000-1-AP. Supplier validation: "Reactivity: Human, Mouse, Rat, Applications: WB, IP, IHC, IF, ELISA".
Anti-human LC3B antibody Novus, Cat# NB100-2220. Supplier validation: "Reactivity Hu, Mu, Rt, Po, Al, Av, Ba, Bv, Ca, Ch, ChHa, Gp, Ha, In, Pm, Pm, Rb, SyHa, Ze, Applications WB, Simple Western, ELISA, Flow, Func, ICC/IF, IHC, IP, PAGE, WB, ChIP".
Brilliant Violet 421 anti-human CD206 antibody Biolegend, Cat# 321125. Supplier validation: "Verified Reactivity Human, Application FC - Quality tested".

PerCP/Cyanine5.5 anti-mouse CD45 antibody Biolegend, Cat# 103132. Supplier validation: "Verified Reactivity Mouse, Application FC - Quality tested".
Brilliant Violet 785 anti-mouse CD3 antibody Biolegend, Cat# 100232. Supplier validation: "Verified Reactivity Mouse, Application FC - Quality tested".
FITC anti-mouse CD4 antibody Biolegend, Cat# 100509. Supplier validation: "Verified Reactivity Mouse, Application FC - Quality tested".
Alexa Fluor® 700 anti-mouse CD8 antibody Biolegend, Cat# 100730. Supplier validation: "Verified Reactivity Mouse, Application FC - Quality tested".
PE anti-mouse IFN-γ antibody Biolegend, Cat# 505808. Supplier validation: "Verified Reactivity Mouse, Application FC - Quality tested".
Brilliant Violet 605™ anti-mouse Ki67 antibody Biolegend, Cat# 652413. Supplier validation: "Verified Reactivity Mouse, Application FC - Quality tested".
PE anti-mouse F4/80 antibody Biolegend, Cat# 123110. Supplier validation: "Verified Reactivity Mouse, Application FC - Quality tested".
PE/Cyanine7 anti-mouse CD11b antibody Biolegend, Cat# 101216. Supplier validation: "Verified Reactivity Mouse, Application FC - Quality tested".
Alexa Fluor® 647 anti-mouse CD206 antibody Biolegend, Cat# 141712. Supplier validation: "Verified Reactivity Mouse, Application FC - Quality tested".
Brilliant Violet 605™ anti-mouse PD-1 antibody Biolegend, Cat# 135220. Supplier validation: "Verified Reactivity Mouse, Application FC - Quality tested".
PE/Cyanine7 anti-mouse TIM3 antibody Biolegend, Cat# 119716. Supplier validation: "Verified Reactivity Mouse, Application FC - Quality tested".
PE anti-mouse LAG-3 antibody Biolegend, Cat# 125208. Supplier validation: "Verified Reactivity Mouse, Application FC - Quality tested".
Brilliant Violet 421™ anti-mouse CD25 antibody Biolegend, Cat# 102043. Supplier validation: "Verified Reactivity Mouse, Application FC - Quality tested".
Alexa Fluor® 647 anti-mouse Foxp3 antibody Biolegend, Cat# 320014. Supplier validation: "Verified Reactivity Mouse, Application FC - Quality tested".
PE anti-mouse CD152 antibody Biolegend, Cat# 106306. Supplier validation: "Verified Reactivity Mouse, Application FC - Quality tested".
Zombie Aqua Biolegend, Cat# 423102. Supplier validation: "Application, FC, ICFC - Quality tested, ICC - Verified".
Anti-mouse CD3ε antibody, functional grade Biolegend, Cat# 100340. Supplier validation: "Verified Reactivity Mouse, Application FC - Quality tested, IHC-F, IP, Activ, Block, WB, ICC ".
Anti-mouse CD28 antibody, functional grade Biolegend, Cat# 102116. Supplier validation: "Verified Reactivity Mouse, Application FC - Quality tested, IP, IHC-F, Costim, Block ".
Anti-mouse IL-1 beta antibody Thermo Scientific, Cat# 14-7012-81. Supplier validation: "Verified Reactivity Mouse, Rat, Application FC, ELISA, Neu, FN".
Anti-mouse LAG-3 antibody BioXcell, Cat# BP0174. Supplier validation: "Verified Reactivity Mouse, Reported Applications in vivo LAG-3 neutralization, in vitro LAG-3 neutralization, Flow cytometry, Western blot".
Anti-mouse TNFα antibody Biolegend, Cat# 506111. Supplier validation: "Verified Reactivity Mouse, Rat, Application ELISA Capture - Quality tested, ELISPOT Capture, Neut, ICFC, IP, WB".
Anti-mouse CSF1R antibody Bioxcell, Cat# BE0213. Supplier validation: "Reported Applications in vivo macrophage depletion, in vitro CSF1R neutralization, in vivo monocyte depletion, Flow cytometry, Western blot".

Eukaryotic cell lines

Policy information about cell lines and Sex and Gender in Research

Cell line source(s)	B16F10, CT26, 293T, and HT29 cells were purchased from American Type Culture Collection (ATCC, Manassas, USA). The MC38 (mouse colon adenocarcinoma) cell line was kindly gifted by Prof. Wei Yang (Guangdong Provincial People's Hospital). MEF WT, MEF p65-/-, and MEF IKK-/- cells were kindly provided by Dr. Paul J. Chiao's laboratory (MD Anderson Cancer Center, USA).
Authentication	All cell lines were authenticated based on STR fingerprinting before use.
Mycoplasma contamination	All cell lines were tested negative for mycoplasma contamination. Stated in the Methods section.
Commonly misidentified lines (See ICLAC register)	No commonly misidentified cell lines were used.

Animals and other research organisms

Policy information about studies involving animals; ARRIVE guidelines recommended for reporting animal research, and Sex and Gender in Research

Laboratory animals	All mice used in this study were C57BL/6J female mice (6 weeks), which were purchased from Beijing Vital River Laboratories (Beijing, China), Rag1-/- (Strain NO. T004753) female mice (5-6 weeks) and NOD/ShiLtJGpt-Prkdcem26Cd52Il2rgem26Cd22/Gpt (NCG, Strain NO. T001475) female mice (5-6 weeks) that were purchased from Gempharmatech (Jiangsu, China). Mice were housed in temperature-controlled pathogen-free conditions (around 20℃, 40% humidity) under a 12-h light/dark cycle.

Wild animals	The study did not involve wild animals
Reporting on sex	Sex is not relevant in this study. Only female mice were used in this study.
Field-collected samples	This study did not include field-collected samples.
Ethics oversight	All animal studies were performed in accordance with a protocol approved by the Institutional Ethics Committee for Clinical Research and Animal Trials of the Sun Yat-sen University Cancer Center (L025501202112009).

Note that full information on the approval of the study protocol must also be provided in the manuscript.

Flow Cytometry

Plots

Confirm that:

- [x] The axis labels state the marker and fluorochrome used (e.g. CD4-FITC).
- [x] The axis scales are clearly visible. Include numbers along axes only for bottom left plot of group (a 'group' is an analysis of identical markers).
- [x] All plots are contour plots with outliers or pseudocolor plots.
- [x] A numerical value for number of cells or percentage (with statistics) is provided.

Methodology

Sample preparation	Fresh isolated tumor samples from liver metastases were minced into small pieces using a scalpel, digested with a Tumor Dissociation Kit (Miltenyi Biotec, Bergisch Gladbach, Germany) and incubated at 37 °C with rotation for 30 min. The samples were passed through 70 µm and 40 µm nylon cell strainers and washed with FACS buffer (PBS supplemented with 2% FBS). After removal of the supernatant, the remaining cells were gently suspended in 40% Percoll (Cytiva, Marlborough USA) and purified by gradient centrifugation for 20 min. The remaining cells were resuspended in FACS buffer, stained with Zombie, anti-mouse CD45, CD3, CD4, CD8, IFN-γ and/or Ki67 antibodies and evaluated by flow cytometry.
Instrument	CytoFLEX LX (Beckman Coulter)
Software	CytExpert software (Beckman Coulter); FlowJo version 10
Cell population abundance	Reported in Supplementary information and Method section.
Gating strategy	Reported in Supplementary information.

- [x] Tick this box to confirm that a figure exemplifying the gating strategy is provided in the Supplementary Information.
